# Crystal structure of 2,4-di-*tert*-butyl-6-(hy­droxy­methyl)­phenol

**DOI:** 10.1107/S2056989016016753

**Published:** 2016-10-25

**Authors:** Ane I. Aranburu Leiva, Sophie L. Benjamin, Stuart K. Langley, Ryan E. Mewis

**Affiliations:** a School of Science and the Environment, Division of Chemistry and Environmental Science, Manchester Metropolitan University, John Dalton Building, Chester St, Manchester, M1 5GD, England; b School of Science and Technology, Nottingham Trent University, Nottingham, NG11 8NS, England

**Keywords:** crystal structure, O—H⋯O hydrogen bonding, intra- and inter­molecular hydrogen bonding, pendent arm

## Abstract

The title compound, C_15_H_24_O_2_, is an example of a phenol-based pendant-arm precursor. In the mol­ecule, the phenol hy­droxy group participates in an intra­molecular O—H⋯O hydrogen bond with the pendant alcohol group, forming an *S*(6) ring. This ring adopts a half-chair conformation. In the crystal, O—H⋯O hydrogen bonds connect mol­ecules related by the 3_1_ screw axes, forming chains along the *c* axis. The C—C—O angles for the hy­droxy groups are different as a result of the type of hybridization for the C atoms that are involved in these angles. The C—C—O angle for the phenol hy­droxy group is 119.21 (13)°, while the angle within the pendant alcohol is 111.99 (13)°. The bond length involving the phenolic oxygen is 1.3820 (19) Å, which contrasts with that of the alcoholic oxygen which is 1.447 (2) Å. The former is conjugated with the aromatic ring and so leads to the observed shorter bond length.

## Abstract 2

The crystal structure of 2,4-di-*tert*-butyl-6-hy­droxy­methyl­phenol is presented.

## Chemical context   

The addition of pendent arms to ligands, which possess donor atoms that are capable of ligating to a metal ion, aid the stabilization of the resulting complex formed. In particular, the use of phenol-based ligands are of inter­est because they are used to form stable phenoxyl radicals, which are found in some enzymatic active sites, such as photosystem II and galactose oxidase (Rogers & Dooley, 2003 ▸; Pujols-Ayala & Barry, 2004 ▸). Synthesis of pendent arms containing phenolate moieties have been used for the creation of biomimetic complexes and for the study of their redox properties (Zhu *et al.*, 1996 ▸; Kimura *et al.*, 2001 ▸; Esteves *et al.*, 2013 ▸; Sokolowski *et al.*, 1997 ▸). The creation of pendent arms that possess functional groups, which can be easily manipulated to give possible tethering points (such as the transformation of an alcohol to the corresponding alkyl halide), or groups that are easily protected to prevent unwanted side reactions are, therefore, highly desirable.
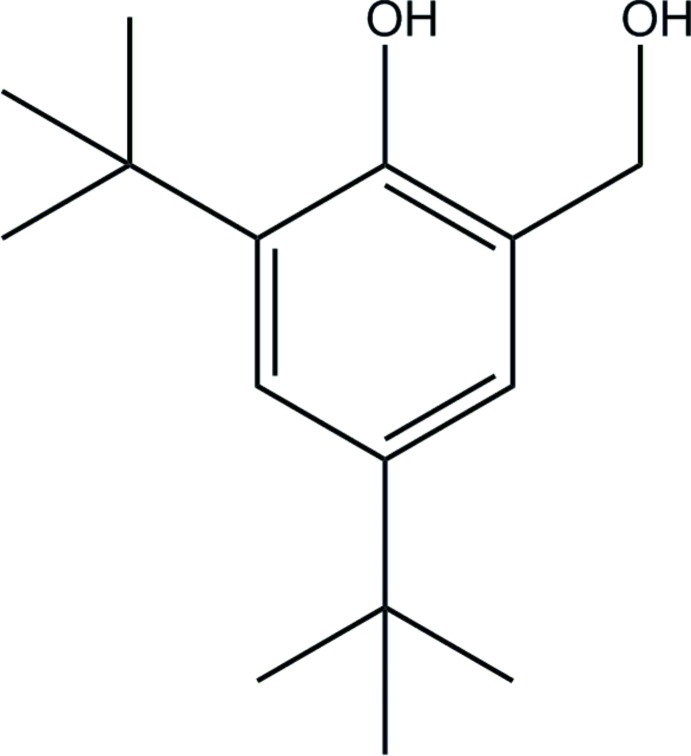


As part of our work on the synthesis of macrocyclic ligand systems bearing phenolate pendent arms, we report the crystal structure of 2,4-di-*tert*-butyl-6-hy­droxy­methyl­phenol, (I), which is an inter­mediary in a pendent-arm synthesis.

## Structural commentary   

The mol­ecule of (I) possesses an intra­molecular hydrogen bond (Table 1 ▸). This inter­action does not cause any sizable deviation from the idealized bond angle, as the bond angle for C6—C15—O2 is 111.99 (13)°, whilst the bond angle for C6—C1—O1 is 119.21 (13)°. Furthermore, the formation of an intra­molecular hydrogen bond within the structure creates a six-membered ring system that involves C1, C6, C15, O2, H1*O*1 and O1. This six-membered ring has a half-chair conformation. The phenolic C—O bond length is 1.3820 (19) Å, which is shorter than the alcoholic C—O bond length [1.447 (2) Å] due to conjugation with the aromatic ring. The aromatic ring is planar, as expected, and has inter­nal bond angles that range from 116.49 (14) to 123.95 (14)°. The bond lengths from the quaternary atoms of the *tert*-butyl group to the nearest aromatic ring carbon are very similar (the average bond length is 1.54 Å).

## Supra­molecular features   

**Figure 1:**
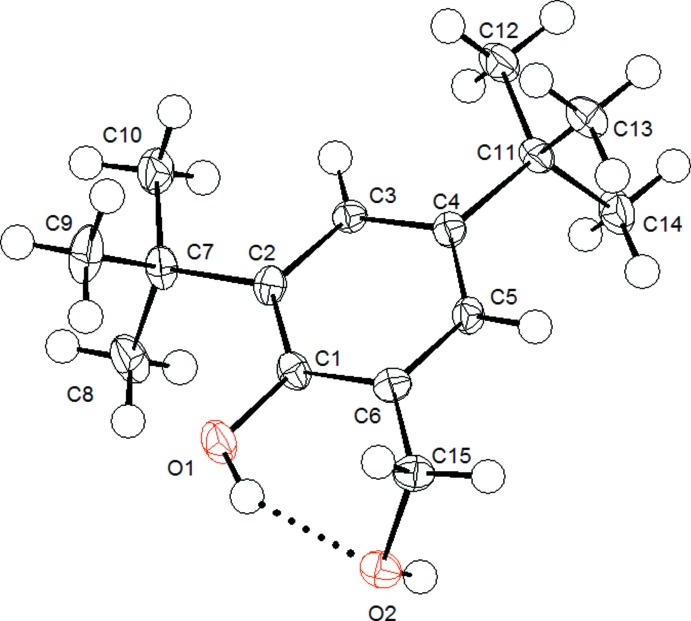
The mol­ecular structure of compound (I), showing the atom labelling and displacement ellipsoids drawn at the 50% probability level. The intra­molecular hydrogen bond is shown by the dashed bond.

**Figure 2:**
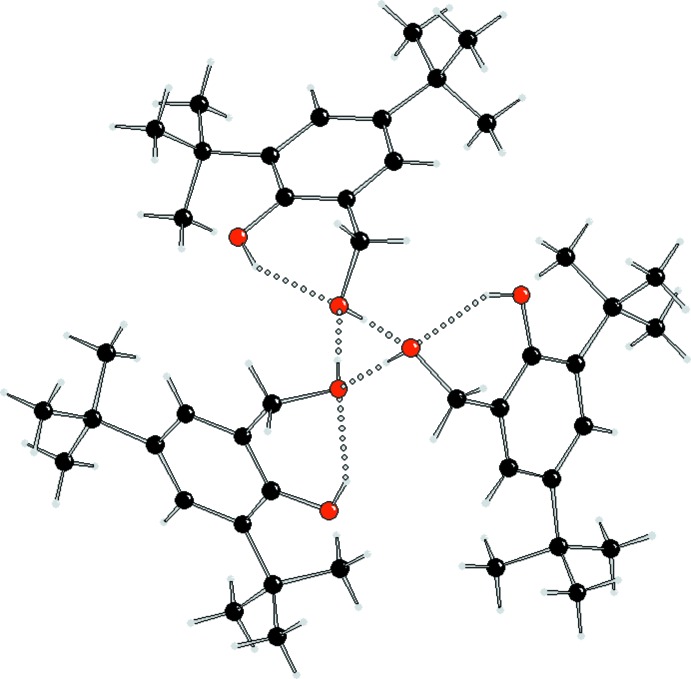
The crystal packing of compound (I), viewed along the *c-*axis direction. The hydrogen bonds are shown as dashed lines.

In the crystal structure of (I) (Fig. 1 ▸), mol­ecules are linked by inter­molecular hydrogen bonds that are much shorter than the intra­molecular hydrogen bonds (see Table 1 ▸). Inter­molecular hydrogen bonds are formed between mol­ecules that are related by a 3_1_ screw axis which generates chains along the *c*-axis direction (Figs. 2 ▸ and 3 ▸). The inter­molecular hydrogen bond is stronger than the intra­molecular bond due to collinearity between the proton donor group (O2—H1*O*2) and the proton acceptor (O2^i^). The bond angle for O2—H1*O*2⋯O2^i^ is 178 (2)°, which contrasts strongly with the weaker intra­molecular hydrogen bond, which is 146 (2)° (O1—H1*O*1⋯O2). The presence of inter­molecular hydrogen bonding is the only inter­action that stabilizes the 1D structure, as there are no π–π stacking inter­actions present; the aromatic rings are separated by more than 6 Å.

## Database survey   

A search of the Cambridge Structural Database (Version 5.37, update February 2016; Groom *et al.*, 2016 ▸) for the substructure of 2,4-di-*tert*-butyl-6-hy­droxy­methyl­phenol yielded 29 hits (the carbon of the CH_2_ group was restricted to have a coordination of four atoms, and the phenolic oxygen two atoms). Of these 29 hits, 14 were organic compounds; the remainder were all metal complexes. A number of compounds used the same mol­ecular motif to form ethers *via* the alcoholic oxygen [AVOPOR and AVOQET (Huang *et al.*, 2010 ▸); BERLIV, BERLOB, BURLAH and BERMAO (Huang *et al.*, 2013 ▸); WUZJAE and WUZHOW (Audouin *et al.*, 2015 ▸)]. A further sub-set of inter­est was where the two hydrogen atoms of the CH_2_ group of (I) have been replaced by CF_3_/C_6_F_5_ groups to coordinate to titanium(IV) centres [ZUNWOW and ZUNWUC (Tuskaev *et al.*, 2015 ▸); XEMBAU and XEMREY (Solov’ev *et al.*, 2011 ▸)]. ZUNWOW is noteworthy because fluorine also acts as a ligand to a coordinated lithium ion. Two oxazole structures that contain the title compound were also identified [KUTQUM (Campbell *et al.*, 2010 ▸); LUYSIU (Błocka *et al.*, 2010 ▸)], although neither used (I) as a starting material. The only structure that utilizes 2,4-di-*tert*-butyl-6-hy­droxy­methyl­phenol without modification is a complex that contains two titanium(IV) centres, four 2,4-di-*tert*-butyl-6-hy­droxy­methyl­phenol ligands and two chloride ligands (BAFFOG; Gagieva *et al.*, 2014 ▸). Two of the 2,4-di-*tert*-butyl-6-hy­droxy­methyl­phenol ligands display bridging through the alcoholic oxygen to both Ti^IV^ centres. The C—O bond lengths are comparable to those of (I); the phenolic C—O bond length in BAFFOG shows the largest difference in that it contracts by 0.015 Å relative to (I). Furthermore, the bond lengths of the six-membered ring that is formed between the ligand and the Ti^IV^ centre also closely resembles that of (I); the only noteworthy difference between the two structures are the two bond lengths that involve oxygen to either Ti^IV^ or H1*O*1. In the former they are 2.003 and 1.832 Å whereas in (I) they are 2.03 (2) and 0.84 (2) Å.

## Synthesis and crystallization   

The synthesis of 2,4-di-*tert*-butyl-6-hy­droxy­methyl­phenol is based on a reported literature procedure (Wang *et al.*, 2014 ▸). 2,4-Di-*tert-*butyl­phenol (5 g, 0.024 mol) and LiOH·H_2_O (0.083 g, 0.002 mol) were dissolved in methanol (10 mL), and a suspension of paraformaldehyde (4.50 g, 0.15 mol) in methanol (10 mL) was added at room temperature. The reaction mixture was heated to reflux for 24 hr. After being allowed to cool to room temperature, the solvent was removed under reduced pressure and the white residue was dissolved in diethyl ether. The organic layer was washed with water (3 x 50 mL). The organic layer was collected and dried with magnesium sulfate. The solvent was removed by rotary evaporation to yield a white powder (2.3 g, 40%). Part of the purified product was re-dissolved in *n*-hexane and placed in a refrigerator. After several days, colourless needle-like crystals were obtained. ^1^H NMR (CDCl_3_, 400 MHz): δ 7.55 (*s*, 1H, CH_2_OH), 7.28 (*d*, 1H, *J =* 2.52 Hz, ArH), 6.89 (*d*, 1H, *J =* 2.52 Hz, ArH), 4.84 (*s*, 2H, CH_2_OH), 1.41 (*s*, 9H, ^*t*^Bu), 1.29 (*s*, 9H, ^*t*^Bu); ^13^C NMR (CDCl_3_, 100 MHz): δ 153.21, 141.69, 136.60, 124.19, 124.04, 122.70 (C_arom_), 66.00 (CH_2_), 35.04, 34.30 [C(^*t*^Bu)], 31.69, 29.75 [Me(^*t*^Bu)]. IR (KBr pellet, cm^−1^): 3530 (*w*), 3424 (*w*), 3175 (*w*, *br*), 2954 (*s*), 2905 (*s*), 2866 (*m*), 1067 (*w*), 1506 (*s*), 1481 (*s*), 1463 (*s*), 1445 (*s*), 1417 (*m*), 1391 (*s*), 1361 (*s*), 1301 (*w*), 1278 (*w*), 1250 (*w*), 1227 (*s*), 1201 (*s*), 1163 (*w*), 1125 (*m*), 1084 (*w*), 1026 (*s*), 942 (*s*), 927 (*s*), 879 (*s*), 823 (*m*), 797 (*m*), 763 (*m*), 723, (*m*), 654 (*m*).

## Refinement details   

Crystal data, data collection and structure refinement details are summarized in Table 2 ▸. All hydrogen atoms are placed in calculated positions [C—H = 0.98–0.99Å; *U*_iso_(H) = 1.2 or 1.5*U*_eq_(C)], except for H1*O*1 and H1*O*2 which were located in a difference map and their positions freely refined with *U*_iso_(H) = 0.05 for both. The absolute structure could not be determined from the X-ray data.

## Supplementary Material

Crystal structure: contains datablock(s) I. DOI: 10.1107/S2056989016016753/lh5825sup1.cif

Structure factors: contains datablock(s) I. DOI: 10.1107/S2056989016016753/lh5825Isup2.hkl

Supporting information file. DOI: 10.1107/S2056989016016753/lh5825Isup3.cml

CCDC reference: 1510636

Additional supporting information: crystallographic information; 3D view; checkCIF report

## Figures and Tables

**Figure 3:**
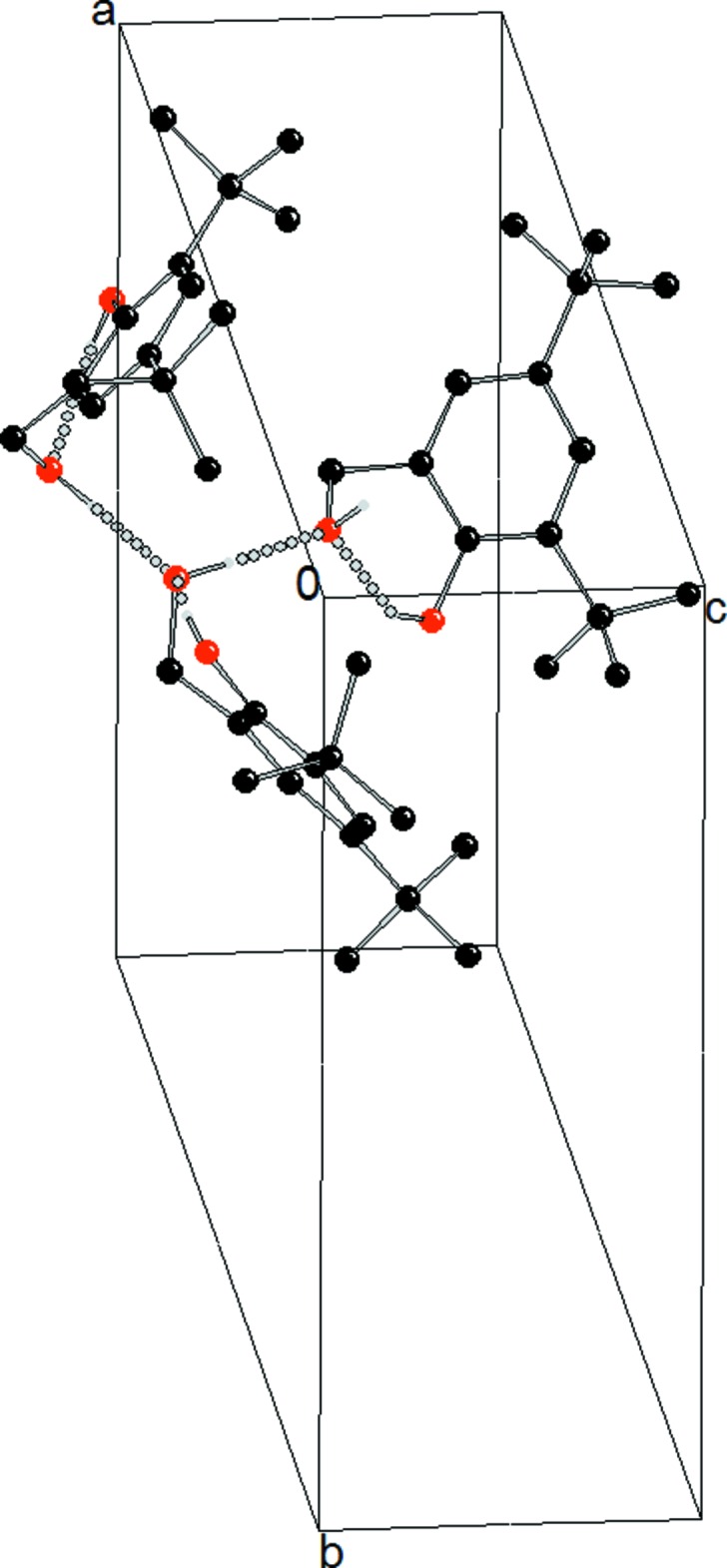
The crystal packing of compound (I) showing the helical chains along the *c* axis. Hydrogen bonds are shown as dashed lines.

**Table 1:** Hydrogen-bond geometry (Å, °)

*D*—H⋯*A*	*D*—H	H⋯*A*	*D*⋯*A*	*D*—H⋯*A*
O2—H1*O*2⋯O2^i^	0.91 (2)	1.75 (2)	2.6636 (14)	178 (2)
O1—H1*O*1⋯O2	0.84 (2)	2.03 (2)	2.7706 (18)	146 (2)

Symmetry code: (i) 
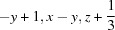
.

**Table 2:** Experimental details

Crystal data	
Chemical formula	C_15_H_24_O_2_
*M*_r_	236.34
Crystal system, space group	Trigonal, *P*3_1_
Temperature (K)	123
*a*, *c* (Å)	14.4357 (9), 6.0404 (5)
*V* (Å^3^)	1090.11 (13)
*Z*	3
Radiation type	Mo *K*α
μ (mm^−1^)	0.07
Crystal size (mm)	0.5 × 0.1 × 0.05

Data collection	
Diffractometer	Agilent Xcalibur
Absorption correction	Multi-scan (*CrysAlis PRO*; Agilent, 2014 ▸)
*T*_min_, *T*_max_	0.992, 0.997
No. of measured, independent and observed [*I* > 2σ(*I*)] reflections	6245, 3097, 2883
*R*_int_	0.025
(sin θ/λ)_max_ (Å^−1^)	0.649

Refinement	
*R*[*F* ^2^ > 2σ(*F* ^2^)], *wR*(*F* ^2^), *S*	0.042, 0.089, 1.08
No. of reflections	3097
No. of parameters	166
No. of restraints	1
H-atom treatment	H atoms treated by a mixture of independent and constrained refinement
Δρ_max_, Δρ_min_ (e Å^−3^)	0.20, −0.22

Computer programs: *CrysAlis PRO* (Agilent, 2014 ▸), *SHELXS97* and *SHELXL97* (Sheldrick, 2008 ▸), *X-SEED* (Barbour, 2001 ▸) and *CAMERON* (Watkin *et al.*, 1996 ▸).

## supplementary crystallographic information

### Crystal data

**Table:**

C_15_H_24_O_2_	*D*_x_ = 1.080 Mg m^−^^3^
*M**_r_* = 236.34	Mo *K*α radiation, λ = 0.71073 Å
Trigonal, *P*3_1_	Cell parameters from 9833 reflections
*a* = 14.4357 (9) Å	θ = 3.1–27.5°
*c* = 6.0404 (5) Å	µ = 0.07 mm^−^^1^
*V* = 1090.11 (13) Å^3^	*T* = 123 K
*Z* = 3	Needle, colourless
*F*(000) = 390	0.5 × 0.1 × 0.05 mm

### Data collection

**Table:**

Agilent Xcalibur diffractometer	3097 independent reflections
Radiation source: fine-focus sealed tube	2883 reflections with *I* > 2σ(*I*)
Graphite monochromator	*R*_int_ = 0.025
Detector resolution: 15.9832 pixels mm^-1^	θ_max_ = 27.5°, θ_min_ = 3.3°
scans in φ and ω	*h* = −18→17
Absorption correction: multi-scan (CrysAlisPro; Agilent, 2014)	*k* = −17→18
*T*_min_ = 0.992, *T*_max_ = 0.997	*l* = −7→7
6245 measured reflections	

### Refinement

**Table:**

Refinement on *F*^2^	Primary atom site location: structure-invariant direct methods
Least-squares matrix: full	Secondary atom site location: difference Fourier map
*R*[*F*^2^ > 2σ(*F*^2^)] = 0.042	Hydrogen site location: inferred from neighbouring sites
*wR*(*F*^2^) = 0.089	H atoms treated by a mixture of independent and constrained refinement
*S* = 1.08	*w* = 1/[σ^2^(*F*_o_^2^) + (0.0403*P*)^2^ + 0.0827*P*] where *P* = (*F*_o_^2^ + 2*F*_c_^2^)/3
3097 reflections	(Δ/σ)_max_ = 0.001
166 parameters	Δρ_max_ = 0.20 e Å^−^^3^
1 restraint	Δρ_min_ = −0.22 e Å^−^^3^

### Special details

**Table:**

Geometry. All esds (except the esd in the dihedral angle between two l.s. planes) are estimated using the full covariance matrix. The cell esds are taken into account individually in the estimation of esds in distances, angles and torsion angles; correlations between esds in cell parameters are only used when they are defined by crystal symmetry. An approximate (isotropic) treatment of cell esds is used for estimating esds involving l.s. planes.
Refinement. Refinement of F^2^ against ALL reflections. The weighted R-factor wR and goodness of fit S are based on F^2^, conventional R-factors R are based on F, with F set to zero for negative F^2^. The threshold expression of F^2^ > 2sigma(F^2^) is used only for calculating R-factors(gt) etc. and is not relevant to the choice of reflections for refinement. R-factors based on F^2^ are statistically about twice as large as those based on F, and R- factors based on ALL data will be even larger.

### Fractional atomic coordinates and isotropic or equivalent isotropic displacement parameters (Å^2^)

**Table:**

	*x*	*y*	*z*	*U*_iso_*/*U*_eq_	
O2	0.70639 (10)	0.41386 (10)	−0.00481 (18)	0.0260 (3)	
C3	0.73856 (12)	0.70473 (12)	0.5036 (2)	0.0199 (3)	
H3	0.7539	0.7548	0.6196	0.024*	
C4	0.63425 (12)	0.64968 (12)	0.4213 (2)	0.0204 (3)	
O1	0.87583 (9)	0.59921 (11)	0.1671 (2)	0.0314 (3)	
C6	0.69423 (12)	0.56297 (12)	0.1580 (2)	0.0218 (3)	
C2	0.82219 (12)	0.69053 (12)	0.4252 (2)	0.0216 (3)	
C1	0.79714 (12)	0.61721 (13)	0.2511 (2)	0.0223 (3)	
C11	0.54260 (12)	0.66266 (13)	0.5165 (3)	0.0228 (3)	
C5	0.61420 (12)	0.57839 (13)	0.2466 (3)	0.0219 (3)	
H5	0.5440	0.5395	0.1871	0.026*	
C7	0.93578 (13)	0.75319 (14)	0.5246 (3)	0.0269 (4)	
C15	0.67162 (14)	0.49097 (13)	−0.0400 (3)	0.0260 (4)	
H15A	0.5939	0.4526	−0.0708	0.031*	
H15B	0.7088	0.5352	−0.1711	0.031*	
C13	0.49041 (14)	0.69333 (15)	0.3305 (3)	0.0319 (4)	
H13A	0.4310	0.6999	0.3913	0.048*	
H13B	0.4634	0.6378	0.2159	0.048*	
H13C	0.5436	0.7618	0.2658	0.048*	
C14	0.45901 (13)	0.55618 (14)	0.6205 (3)	0.0323 (4)	
H14A	0.4920	0.5375	0.7414	0.048*	
H14B	0.4322	0.4998	0.5078	0.048*	
H14C	0.3995	0.5633	0.6786	0.048*	
C12	0.58201 (14)	0.74938 (15)	0.6963 (3)	0.0313 (4)	
H12A	0.6358	0.8181	0.6333	0.047*	
H12B	0.6139	0.7300	0.8181	0.047*	
H12C	0.5216	0.7555	0.7522	0.047*	
C8	0.96891 (15)	0.67363 (16)	0.6149 (3)	0.0358 (4)	
H8A	1.0399	0.7134	0.6827	0.054*	
H8B	0.9709	0.6298	0.4929	0.054*	
H8C	0.9169	0.6272	0.7260	0.054*	
C10	0.94193 (14)	0.82504 (15)	0.7178 (3)	0.0348 (4)	
H10A	1.0150	0.8627	0.7767	0.052*	
H10B	0.8923	0.7810	0.8347	0.052*	
H10C	0.9224	0.8773	0.6655	0.052*	
C9	1.01503 (14)	0.82514 (17)	0.3459 (3)	0.0420 (5)	
H9A	0.9933	0.8752	0.2905	0.063*	
H9B	1.0154	0.7807	0.2235	0.063*	
H9C	1.0869	0.8653	0.4097	0.063*	
H1O2	0.6651 (18)	0.3735 (18)	0.111 (4)	0.050*	
H1O1	0.8464 (19)	0.5408 (18)	0.099 (4)	0.050*	

### Atomic displacement parameters (Å^2^)

**Table:**

	*U*^11^	*U*^22^	*U*^33^	*U*^12^	*U*^13^	*U*^23^
O2	0.0308 (6)	0.0292 (6)	0.0208 (6)	0.0170 (5)	0.0048 (5)	0.0005 (5)
C3	0.0212 (7)	0.0180 (7)	0.0200 (7)	0.0095 (6)	0.0007 (6)	0.0012 (6)
C4	0.0197 (7)	0.0198 (8)	0.0213 (8)	0.0095 (6)	0.0013 (6)	0.0040 (6)
O1	0.0226 (6)	0.0407 (8)	0.0319 (7)	0.0166 (6)	0.0016 (5)	−0.0089 (6)
C6	0.0256 (8)	0.0202 (8)	0.0193 (8)	0.0113 (7)	0.0008 (6)	0.0021 (6)
C2	0.0194 (8)	0.0229 (8)	0.0191 (8)	0.0079 (7)	0.0022 (6)	0.0036 (6)
C1	0.0208 (8)	0.0268 (8)	0.0209 (8)	0.0132 (7)	0.0037 (6)	0.0043 (7)
C11	0.0222 (8)	0.0251 (8)	0.0252 (8)	0.0149 (7)	0.0009 (6)	0.0016 (7)
C5	0.0175 (8)	0.0230 (8)	0.0245 (8)	0.0095 (6)	−0.0029 (6)	0.0006 (6)
C7	0.0183 (8)	0.0338 (9)	0.0261 (8)	0.0111 (7)	−0.0005 (6)	−0.0021 (7)
C15	0.0307 (9)	0.0261 (9)	0.0234 (8)	0.0159 (7)	−0.0011 (7)	−0.0014 (7)
C13	0.0314 (9)	0.0392 (11)	0.0341 (9)	0.0243 (8)	−0.0009 (7)	0.0001 (8)
C14	0.0235 (8)	0.0337 (10)	0.0399 (10)	0.0143 (8)	0.0087 (8)	0.0077 (8)
C12	0.0292 (9)	0.0374 (10)	0.0334 (9)	0.0213 (8)	0.0007 (8)	−0.0069 (8)
C8	0.0281 (9)	0.0515 (12)	0.0347 (10)	0.0251 (9)	−0.0056 (8)	−0.0055 (9)
C10	0.0236 (9)	0.0389 (10)	0.0383 (10)	0.0128 (8)	−0.0078 (8)	−0.0103 (8)
C9	0.0214 (9)	0.0470 (12)	0.0398 (10)	0.0038 (8)	0.0029 (8)	0.0002 (9)

### Geometric parameters (Å, º)

**Table:**

O2—C15	1.447 (2)	C15—H15A	0.9900
O2—H1O2	0.91 (2)	C15—H15B	0.9900
C3—C4	1.396 (2)	C13—H13A	0.9800
C3—C2	1.404 (2)	C13—H13B	0.9800
C3—H3	0.9500	C13—H13C	0.9800
C4—C5	1.400 (2)	C14—H14A	0.9800
C4—C11	1.537 (2)	C14—H14B	0.9800
O1—C1	1.3820 (19)	C14—H14C	0.9800
O1—H1O1	0.84 (2)	C12—H12A	0.9800
C6—C5	1.389 (2)	C12—H12B	0.9800
C6—C1	1.405 (2)	C12—H12C	0.9800
C6—C15	1.509 (2)	C8—H8A	0.9800
C2—C1	1.405 (2)	C8—H8B	0.9800
C2—C7	1.544 (2)	C8—H8C	0.9800
C11—C12	1.536 (2)	C10—H10A	0.9800
C11—C13	1.536 (2)	C10—H10B	0.9800
C11—C14	1.536 (2)	C10—H10C	0.9800
C5—H5	0.9500	C9—H9A	0.9800
C7—C10	1.534 (2)	C9—H9B	0.9800
C7—C9	1.538 (2)	C9—H9C	0.9800
C7—C8	1.547 (2)		
			
C15—O2—H1O2	103.7 (14)	H15A—C15—H15B	107.9
C4—C3—C2	123.95 (14)	C11—C13—H13A	109.5
C4—C3—H3	118.0	C11—C13—H13B	109.5
C2—C3—H3	118.0	H13A—C13—H13B	109.5
C3—C4—C5	117.04 (13)	C11—C13—H13C	109.5
C3—C4—C11	123.13 (13)	H13A—C13—H13C	109.5
C5—C4—C11	119.82 (13)	H13B—C13—H13C	109.5
C1—O1—H1O1	108.6 (16)	C11—C14—H14A	109.5
C5—C6—C1	119.30 (14)	C11—C14—H14B	109.5
C5—C6—C15	120.33 (14)	H14A—C14—H14B	109.5
C1—C6—C15	120.35 (14)	C11—C14—H14C	109.5
C3—C2—C1	116.49 (14)	H14A—C14—H14C	109.5
C3—C2—C7	121.49 (14)	H14B—C14—H14C	109.5
C1—C2—C7	122.02 (13)	C11—C12—H12A	109.5
O1—C1—C6	119.21 (13)	C11—C12—H12B	109.5
O1—C1—C2	119.35 (13)	H12A—C12—H12B	109.5
C6—C1—C2	121.44 (13)	C11—C12—H12C	109.5
C12—C11—C13	108.51 (13)	H12A—C12—H12C	109.5
C12—C11—C14	108.18 (14)	H12B—C12—H12C	109.5
C13—C11—C14	109.54 (13)	C7—C8—H8A	109.5
C12—C11—C4	111.91 (13)	C7—C8—H8B	109.5
C13—C11—C4	109.74 (13)	H8A—C8—H8B	109.5
C14—C11—C4	108.92 (13)	C7—C8—H8C	109.5
C6—C5—C4	121.72 (14)	H8A—C8—H8C	109.5
C6—C5—H5	119.1	H8B—C8—H8C	109.5
C4—C5—H5	119.1	C7—C10—H10A	109.5
C10—C7—C9	107.79 (14)	C7—C10—H10B	109.5
C10—C7—C2	112.17 (13)	H10A—C10—H10B	109.5
C9—C7—C2	109.65 (13)	C7—C10—H10C	109.5
C10—C7—C8	107.40 (14)	H10A—C10—H10C	109.5
C9—C7—C8	110.30 (15)	H10B—C10—H10C	109.5
C2—C7—C8	109.51 (14)	C7—C9—H9A	109.5
O2—C15—C6	111.99 (13)	C7—C9—H9B	109.5
O2—C15—H15A	109.2	H9A—C9—H9B	109.5
C6—C15—H15A	109.2	C7—C9—H9C	109.5
O2—C15—H15B	109.2	H9A—C9—H9C	109.5
C6—C15—H15B	109.2	H9B—C9—H9C	109.5

### Hydrogen-bond geometry (Å, º)

**Table:**

*D*—H···*A*	*D*—H	H···*A*	*D*···*A*	*D*—H···*A*
O2—H1*O*2···O2^i^	0.91 (2)	1.75 (2)	2.6636 (14)	178 (2)
O1—H1*O*1···O2	0.84 (2)	2.03 (2)	2.7706 (18)	146 (2)

Symmetry code: (i) −*y*+1, *x*−*y*, *z*+1/3.
